# Draft genome sequence of *Bordetella pertussis* PS21, a circulating clinical isolate from India

**DOI:** 10.1128/mra.00801-25

**Published:** 2025-09-30

**Authors:** Madhumathi Irulappan, Dhivya Murugan, Vishnukumar Ramesh, Subbulakshmi Rajendran, Oliver P. Madhale, Rajeev Zachariah Kompithra, Jobin John Jacob, Balaji Veeraraghavan

**Affiliations:** 1 Department of Clinical Microbiology, Christian Medical College 196380, Vellore, Tamil Nadu, India; 2 Departments of Child Health, Christian Medical College 30025, Vellore, Tamil Nadu, India; DOE Joint Genome Institute, Berkeley, California, USA

**Keywords:** *Bordetella pertussis*, *pertussis* toxin, chemotaxis, virulence determinants

## Abstract

We report the draft genome sequence of *Bordetella pertussis* PS21, a clinical isolate obtained from a culture-positive sample from India. The 3.6 Mb genome harbors classical virulence genes and several notable polymorphisms, including a dual substitution in *ptx*A (Arg5Trp, Met228Ile), a Lys705Glu mutation in *bvg*S, a Lys97 frameshift in *bap*C, and a Gly45Ser substitution in *ptx*B. These mutations may influence pertussis toxin function and virulence regulation, potentially impacting immune recognition by pertussis vaccines.

## ANNOUNCEMENT

Genomic characterization of *Bordetella pertussis* PS21, a clinical isolate from culture-positive sample in India, offers key insights into pertussis pathogenesis and adaptation in low- and middle-income countries. India accounts for ~26% of global pertussis cases (1) yet a culture-positive isolates are scarce, emphasizing the importance of this genomic data set for informing control strategies in a region with high primary vaccination coverage (2).

The PS21 isolate was obtained via nasopharyngeal swab from a 4-week-old child at Christian Medical College, Vellore, India. The child was admitted from 2 to 13 June 2023, with myocarditis and suspected pertussis, treated with azithromycin and later vaccinated with PENTAVAC (DTwP + Hib+ HBV) followed by two doses of QUADROVAX (DPT + Hib). Since the diagnosis preceded vaccination, PS21 likely represents a circulating *B. pertussis* strain. This highlights the risk to unvaccinated neonates and the continued circulation of *B. pertussis* despite vaccination efforts (3).

*B. pertussis PS21* was isolated on Bordet–Gengou agar with 15% defibrinated horse blood and incubated at 35°C for 72 h. Identification was based on morphology and biochemical profile (gram-negative coccobacilli; greyish-white, convex, shiny colonies with “bisected pearl” or “mercury drop” appearance; no growth on MacConkey agar; opaque on charcoal blood agar; catalase/oxidase positive; motility, urease, nitrate reduction, and citrate utilization negative) and confirmed by MALDI-TOF MS (VITEK MS, bioMérieux). Ten colonies were pooled for DNA extraction (Qiagen DNeasy Blood & Tissue Kit).

Libraries (Illumina DNA Prep) were sequenced on the Illumina HiSeq platform (2×151 bp). A total of 740,470 read pairs were obtained. Quality was assessed with FastQC v0.11.9; assembly (SKESA v2.4.0) was evaluated with QUAST (4–6). The 3.6 Mb draft genome comprised 372 contigs (largest 67,885 bp; GC 68%; N50 16,241 bp; N90 5,521 bp) and was annotated using the NCBI Prokaryotic Genome Annotation Pipeline with default parameters (7).

The annotated genome comprises 3,412 protein-coding sequences, 46 tRNA genes, and three rRNA operons. Key virulence genes include the *ptx* operon (*ptx*A-E) (8), *cya*A-E (adenylate cyclase toxin) (9), *dnt* (dermonecrotic toxin) (10), type IV secretion system (*ptl*A-I) (11), type III secretion system (bsc cluster and associated effectors and regulators) (12), fimbrial biogenesis cluster (*fim*A-D, *fim*2, *fim*3, and *fim*X) (13), *fha*C (filamentous hemagglutinin transporter), and complement resistance genes (*bap*C and *brk*A/B) (14).

Comparative genomic analysis of *B. pertussis* strain PS21 was carried out using Snippy v4.6.0, with Tohama I (NC_002929) as the reference genome (15). The analysis included comparisons with six vaccine strains (J445, J446, J447, J448, Bp165, and Pelita III) (16, 17) and six clinical isolates (S1–S5, BPD1, and BPD2) (18). PS21 carried a unique dual substitution in *ptxA* (Arg5Trp and Met228Ile), absent in the compared strains. This mutation may change the structure or stability of the pertussis toxin, possibly affecting its ADP-ribosyltransferase activity (8). Other mutations included Lys705Glu in *bvgS* (global virulence regulator), a Lys97 frameshift in *bapC* (biofilm-associated protein), and Gly45Ser in *ptxB* (pertussis toxin subunit), which were also present in vaccine strains and therefore not unique to PS21 (19).

This genome characterization of PS21 offers insights into *B. pertussis* evolution and supports improved diagnostics and vaccines in resource-limited settings.

## Data Availability

The whole genome has been deposited at NCBI GenBank under accession number JBNCKC000000000, and the corresponding raw sequence reads are deposited in the Sequence Read Archive under accession number SRR33611266.
